# Supplementary material for: Learning fast and fine-grained detection of amyloid neuropathologies from coarse-grained expert labels
Source: bioRxiv. 2023 Jan 17:2023.01.13.524019. Preprint. [Version 1] doi: 10.1101/2023.01.13.524019 (PMC9882138; doi:10.1101/2023.01.13.524019)
Supplement: Supplement 1 [file media-1.pdf]

Supplement for

Learning fast and fine-grained detection of amyloid neuropathologies from coarse-grained expert labels

Daniel R. Wong[1,2,3,4,5], Shino D. Magaki[6], Harry V. Vinters[6,7], William H. Yong[8], Edwin S. Monuki[8], Christopher K. Williams[6], Alessandra C. Martini[8], Charles DeCarli[9], Chris Khacherian[8], John P. Graff[10], Brittany N. Dugger[10*], Michael J. Keiser[1,2,3,4,5*]

1. Institute for Neurodegenerative Diseases, University of California, San Francisco, San Francisco, CA, 94158, USA
2. Bakar Computational Health Sciences Institute, University of California, San Francisco, CA, 94158, USA
3. Department of Pharmaceutical Chemistry, University of California, San Francisco, San Francisco, CA, 94158, USA
4. Department of Bioengineering and Therapeutic Sciences, University of California, San Francisco, San Francisco, CA, 94158, USA
5. Kavli Institute for Fundamental Neuroscience, University of California, San Francisco, San Francisco, CA, 94158, USA
6. Department of Pathology and Laboratory Medicine, University of California, Los Angeles, Los Angeles, CA, 90095, USA
7. Department of Neurology, David Geffen School of Medicine at University of California, Los Angeles, Los Angeles, CA, 90095, USA
8. Department of Pathology & Laboratory Medicine, University of California, Irvine, CA 92697, USA
9. Department of Neurology, School of Medicine, University of California-Davis, Davis, CA 95817, USA
10. Department of Pathology and Laboratory Medicine, School of Medicine, University of California, Davis, Sacramento, CA 95817, USA

*Correspondence: bndugger@ucdavis.edu, keiser@keiserlab.org

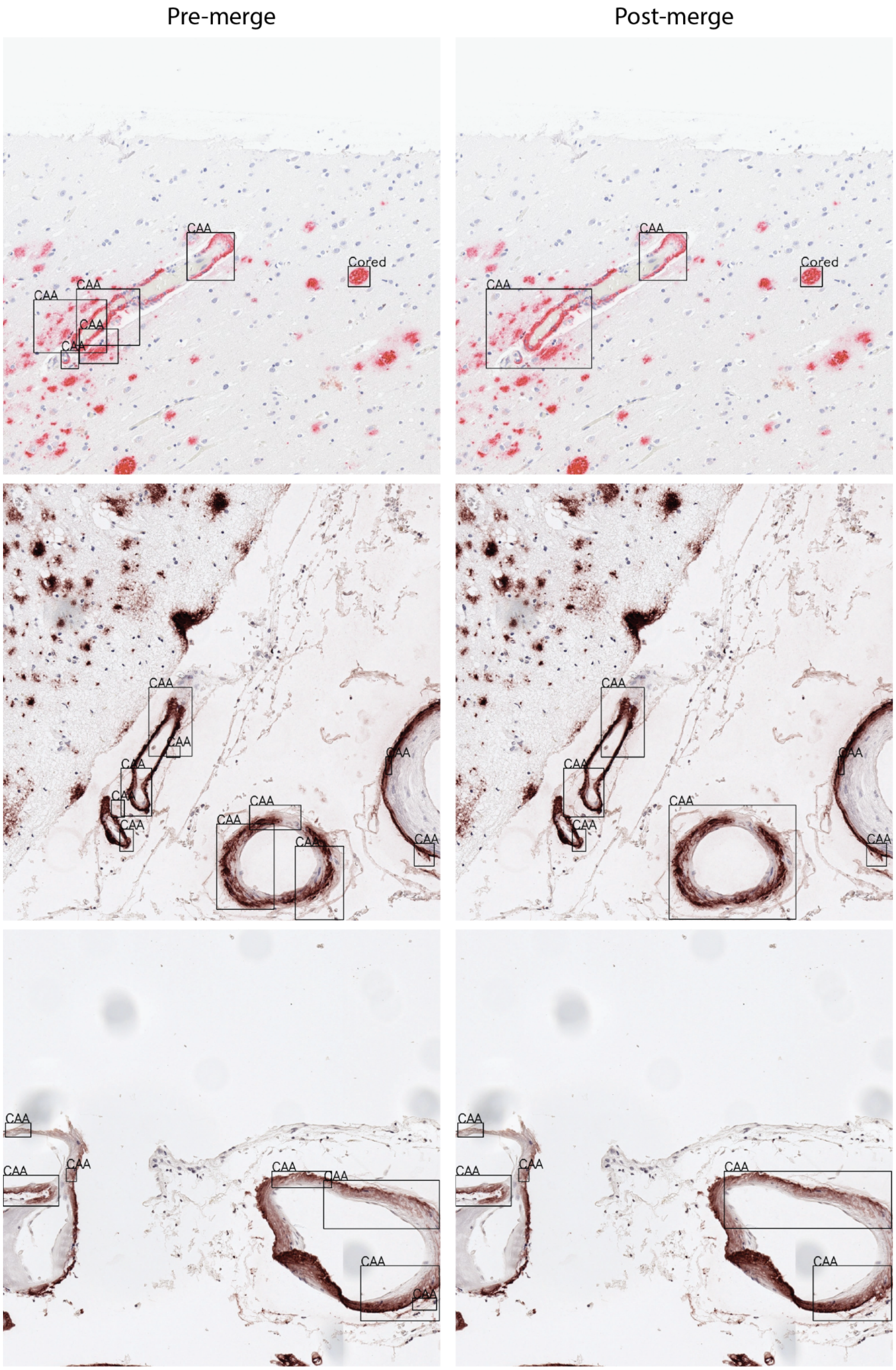

Supplemental Figure 1: Comparing raw pre-merged labels with merged labels. Column left shows the raw bounding box labels. Box coordinates were derived from traditional water-shedding methods unassisted by human intelligence. For each identified class shown, at least two

out of five expert annotators positively labeled the pathology. Column right shows the merged bounding box labels used to train model version one.

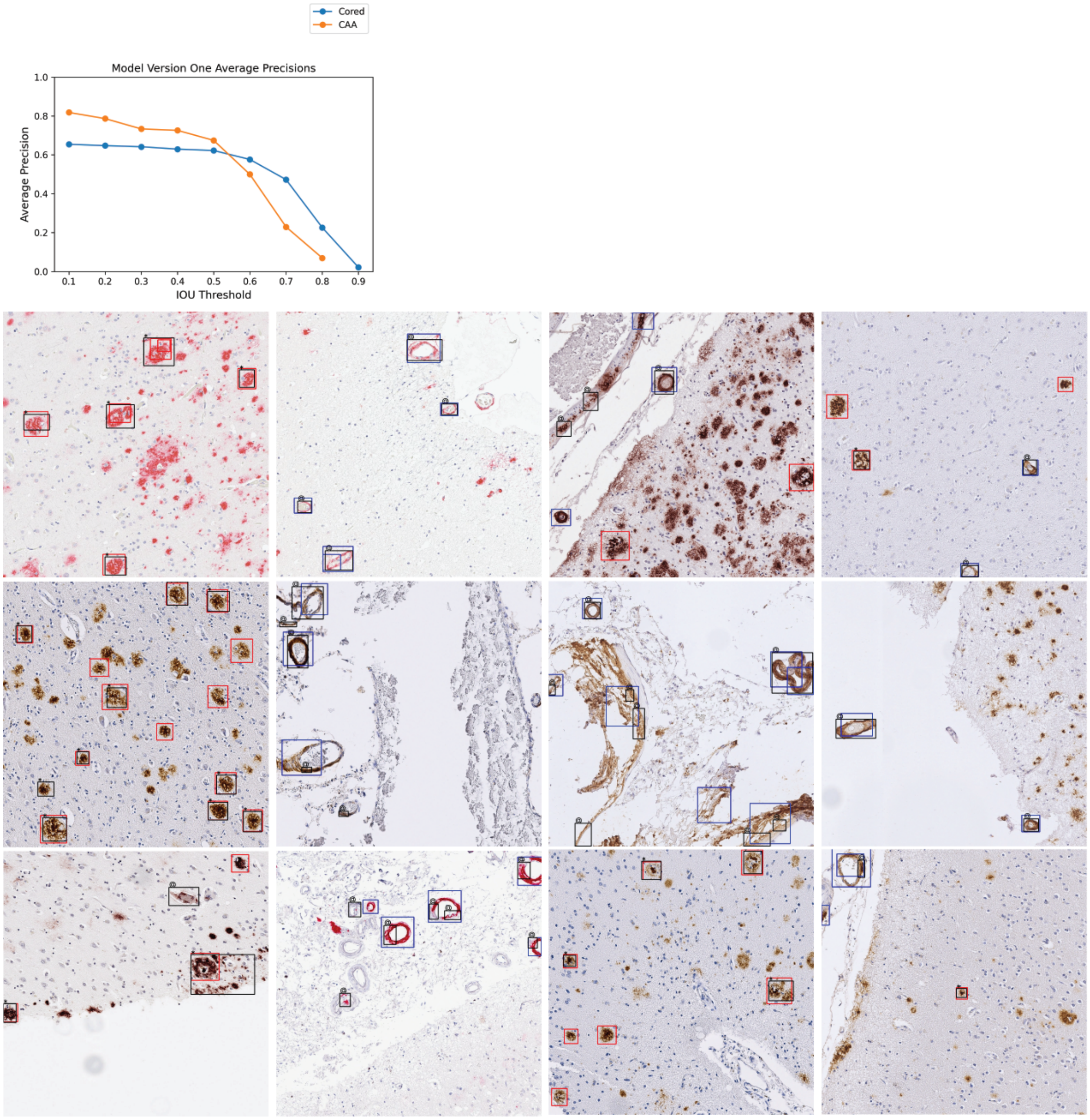

Supplemental Figure 2: Model version one performance and example image predictions. Top: Average precisions over the validation set for various IOU thresholds. The AP at IOU=0.90 is undefined for CAA. Bottom: Example images are pulled from the validation set. Cored prediction: red, Cored label: black "*"; CAA prediction: blue, CAA label: black "@". It is important to note that the label data is sparse and does not contain every pathology (Methods).

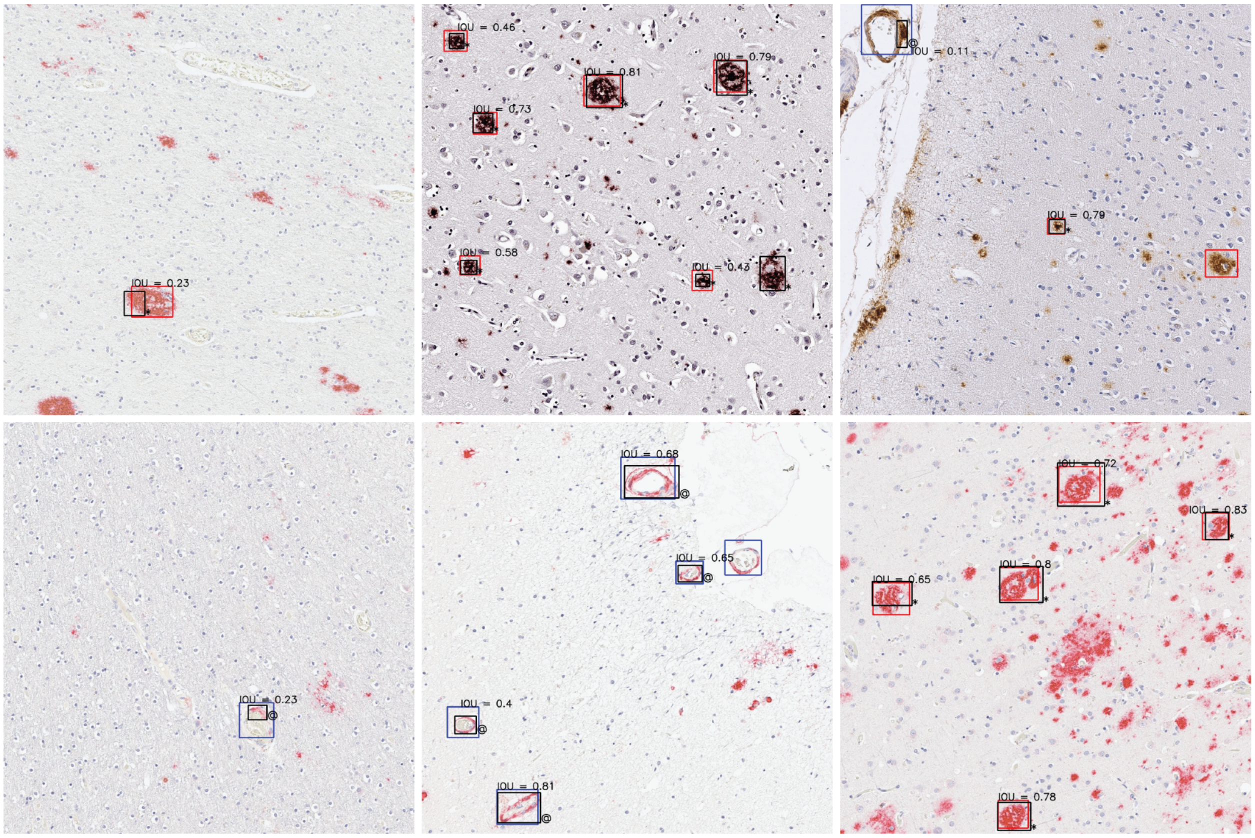

Supplemental Figure 3: Examples of different IOU values for overlaps. IOU values are shown for any overlaps between predicted bounding box (blue for CAA, red for Cored) and label bounding box (black). CAA labels are denoted by the "@" symbol, while Cored labels are denoted by the "*" symbol.

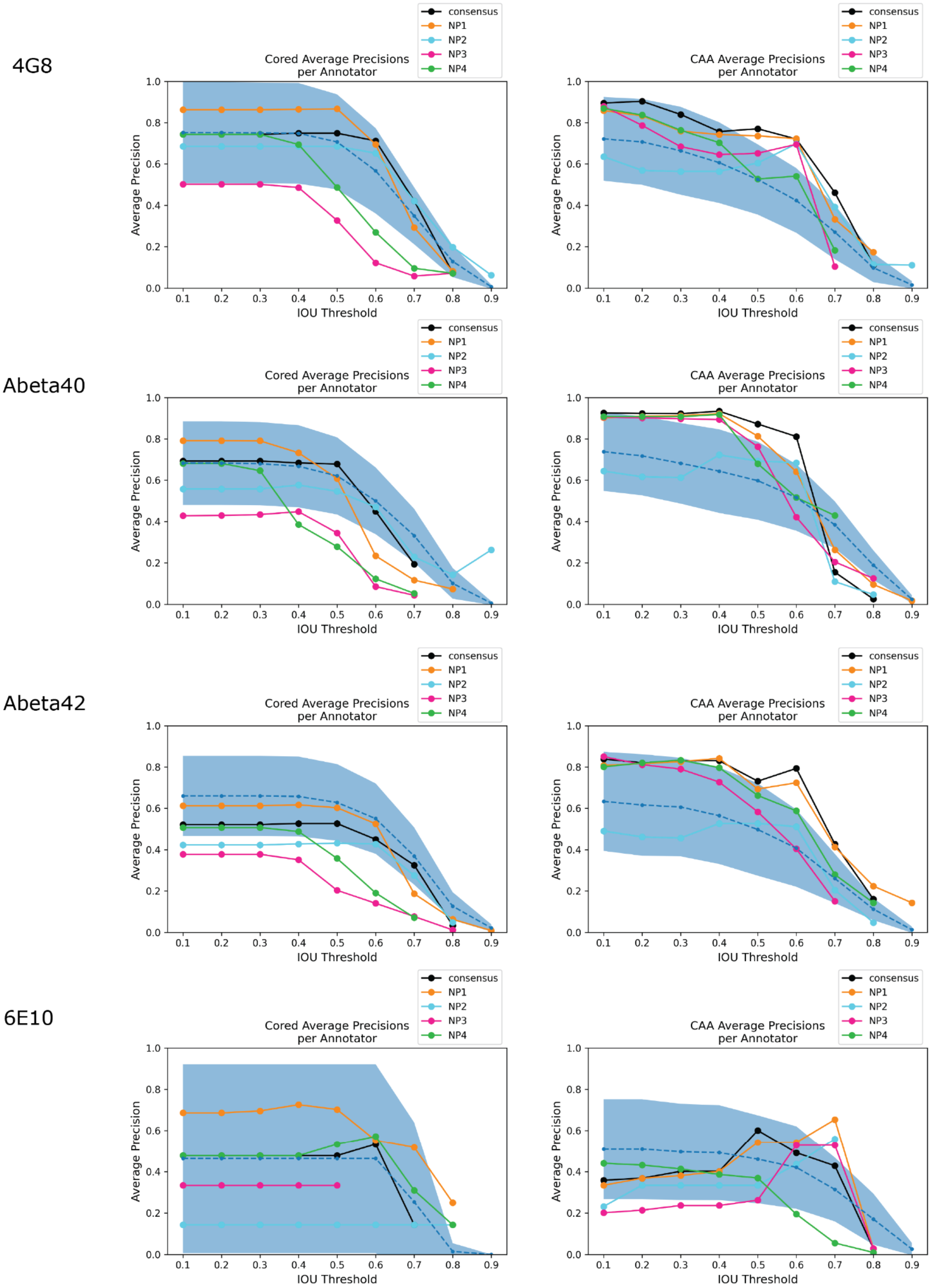

Supplemental Figure 4: Average precision by stain. We compute average precision on the

prospective validation set stratified by stain. At certain IOU thresholds, there are no true positive cases and correspondingly no precision scores.

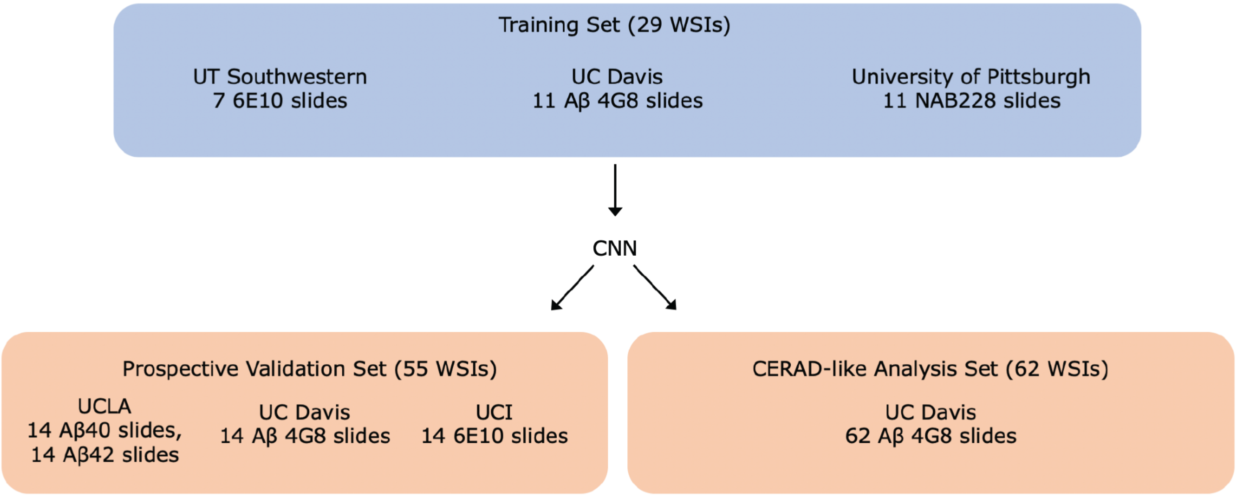

Supplemental Figure 5: Schematic of training and prospective validation datasets. We used a total of 29 WSIs from three institutions for training all models from Wong et al[1]. We used a separate new dataset of 55 WSIs from three institutions for prospective validation. To calculate comparison versus CERAD-like scores (Figure 4), we used a third dataset of 62 WSIs from Tang et al[2].

Architecture	x86_64
CPU op-mode(s)	32-bit, 64-bit
Byte Order	Little Endian
CPUs	64
Thread(s) per core	2
Core(s) per socket	16
Socket(s)	2
NUMA node(s)	2
Vendor ID	GenuineIntel
CPU family	6
Model	79
Model name	Intel(R) Xeon(R) CPU E5-2697A v4 @ 2.60GHz
Stepping	1
CPU MHz	1200.024
CPU max MHz	3600.0000
CPU min MHz	1200.0000
BogoMIPS	5200.02
Virtualization	VT-x
L1d cache	32K

Supplemental Table 1. CPU specifications.

References

1. Wong, D. R. *et al.* Deep learning from multiple experts improves identification of amyloid neuropathologies. *Acta Neuropathol Commun* **10**, 66 (2022).
2. Tang, Z. *et al.* Interpretable classification of Alzheimer's disease pathologies with a convolutional neural network pipeline. *Nat. Commun.* **10**, 2173 (2019).
